# Assessing stress restorative potential of plant species richness and plant landscape types of pocket parks: The mediating role of aesthetic quality

**DOI:** 10.1371/journal.pone.0343001

**Published:** 2026-02-13

**Authors:** Yu Wang, Filzani Illia Ibrahim, Junlin Chang, Siti Norzaini Zainal Abidin

**Affiliations:** 1 Design School, Taylor’s University Lakeside Campus, Subang Jaya, Selangor, Malaysia; 2 School of Architecture Building and Design, Taylor’s University Lakeside Campus, Subang Jaya, Selangor, Malaysia; 3 School of Digital art and Animation, Hebei Institute of Communication, Shijiazhuang City, Hebei Province, China; 4 School of Architecture Building and Design, Taylor’s University Lakeside Campus, Subang Jaya, Selangor, Malaysia; Zhejiang Agriculture and Forestry University: Zhejiang A and F University, CHINA

## Abstract

The issue of stress among urban residents is becoming increasingly serious, affecting both physical and mental health in cities in China. Pocket parks serve as essential green spaces for people’s well-being in high-density urban environments. However, limited empirical research has examined how plant landscape components support stress recovery among urban residents, particularly in the context of pocket parks in China. This study investigated the effects of plant species richness and plant landscape types on stress recovery, with a focus on the mediating role of perceived aesthetic quality. A pre-test and post-test design were conducted in six selected pocket parks with different landscape characteristics using questionnaire surveys. A total of 605 urban residents were recruited using a random sampling method at different sites. The results indicated that medium plant species richness (t = −10.502, *p* < 0.01) and lawn landscape types (t = −12.006, *p* < 0.01) produced the most significant stress recovery effects. Furthermore, perceived aesthetic quality was found to partially mediate the relationship between plant landscape components and stress recovery. The findings of this study contribute to plant landscape research by providing empirical evidence to understand the effects of plant species richness, landscape type, and aesthetic perception on stress recovery in the context of pocket park in China, which is unexplored in past literature.

## 1. Introduction

Urbanization has increased stress for city residents in recent years [1–3]. Chronic stress has severely impacted the physical health and psychological well-being of urban dwellers [4]. An initial nationwide epidemiological survey on mental disorders in China revealed a lifetime prevalence rate of 16.57% among adults with mood disorders, primarily depression and anxiety disorders, showing an increasing trend [5]. Therefore, there is an urgent need for modern urban populations to identify effective methods to alleviate stress among urban population. Urban green spaces play an important role in promoting people’s well-being. Similar concerns have been reported in European and North American cities, where urban green infrastructure has been shown to mitigate mental stress and enhance well-being [6–8].

Pocket parks have been recognized for their critical role in improving urban residents’ well-being. As research on pocket parks has expanded, a general definition of pocket parks has emerged, characterizing as a small-scale green space with discrete distribution, flexible site selection, high utilization rates, and convenient accessibility [9–12]. In this study, pocket parks are defined as independent land use of smaller or diverse forms, with convenient accessibility for residents, possessing certain recreational functions, size from 400m^2^ to 10,000m^2^, and a fragmented spatial distribution within urban areas. This definition is based on ‘Urban Green Space Classification Standard’ issued by the Ministry of Housing and Urban-Rural Development of China (2017).

The linkage between restorative environments and stress recovery is grounded in theories such as Attention Restoration Theory (ART) and Stress Recovery Theory (SRT). ART posits that natural environments help replenish cognitive resources by providing relief from directed attention fatigue through features such as “soft fascination” and “being away” [13]. Meanwhile, SRT emphasizes the role of natural settings in reducing physiological and psychological stress through exposure to calming and non-threatening stimuli [14]. However, most findings stem primarily from studies conducted in large parks or natural reserves, leaving a gap in the literature concerning pocket parks, which are vital for urban populations with limited access to larger natural areas. In addition, most studies have been conducted in a Western context, ignoring the role that cultural context may play in their restorative effects. Evidence from high-density cities in China is limited [15,16].

Aesthetics is an important field of study in landscape design and environmental psychology, emphasizing the perceptual and psychological effects of nature’s interaction with humans [17–19]. However, while the global literature emphasizes the universal benefits of green spaces in promoting mental health, the specific mediating role of perceived aesthetic quality remains insufficiently explored. The basis for aesthetic perception is the immediate, unconscious, and genetically determined emotional reactions—such as fear, aversion, or preference—that occur when the environment is visually exposed [20]. Consequently, these reactions impact later cognitive evaluations of the surroundings, physiological responses, behavior, and overall health. Aesthetic quality involves both objective characteristics of the landscape and subjective perceptions of the observer, making it a complex, multifaceted phenomenon [21]. This duality implies that landscape aesthetics result from an interplay between the interaction between visible environmental attributes and individuals’ psychological processes, including perception, cognition, and emotional responses [20]. Furthermore, vegetation elements within urban green spaces have been shown to influence individuals’ aesthetic perceptions For example, some studies [22,23] have demonstrated a positive correlation between tree planting diversity and aesthetic quality. In addition, recent studies [24,25] have further emphasized the influence of plant richness on aesthetic perceptions.

This study’s findings contribute to bridging this gap by highlighting how aesthetic perceptions dynamically interact with landscape components to influence stress recovery. Overall, this study aims to address these deficiencies by examining the effects of plant species richness and plant landscape type on stress recovery in urban pocket parks. It considers perceived aesthetic quality as a mediating variable. Specifically, two primary research questions are addressed:

(1) What is the effect of different plant species richness (low, medium, and high) and plant landscape types (lawn, tree-shrubs-grass, and tree-grass) on the stress recovery?
(2) What role does the perceived aesthetic quality play in the relationship between plant landscape components and stress recovery?

The following hypotheses were formulated to guide the analysis: H1: Exposure to plant landscapes with higher plant species richness will promote greater stress recovery than those with medium or low biodiversity. H2: Exposure to plant landscapes with lawn landscape will promote greater stress recovery than other landscape types.

## 2. Literature review

### 2.1 Pocket parks as a restorative environment

Pocket parks have gained increasing attention for their potential to serve as restorative environments, particularly in densely populated urban areas. These parks, typically compact in size, are strategically located to provide accessible green spaces to urban dwellers, contributing to improved psychological well-being and enhanced quality of life [9–11]. A growing body of empirical research underscore the restorative benefits of pocket parks. Individuals who spent time in pocket parks reported significant reductions in stress and increased positive mood states [26]. Pocket parks not only provide opportunities for passive relaxation but also support active engagement such as walking and social interaction, which further enhance their restorative effects.

### 2.2 Plant diversity and plant landscape type affecting stress recovery

Landscapes components are crucial element of urban green spaces that provide physical and psychological benefits to humans [4,6,27,28]. Among these components, plant species richness and plant landscape type are important components of the plant landscape in pocket parks that contribute to people’s mental restoration. Consequently, these factors is crucial for pocket park landsacpe design. For example, perceptions of varying plant species richness in campus landscapes and their subsequent restorative effects were evaluated among university students [29]. A study in US considered general traits, such as tree size and shape, but neglected plant species richness, a key factor determining the quality of green spaces [30]. A previous qualitative study used semi-structured interviews to explore the complex relationship between perceived biodiversity and stress recovery potential in forests area of southern Finland [31]. Past findings emphasized the potential of biodiversity nature to improve well-being across the board. However, to date, there is still a lack of empirical evidence in studies on different plant diversity levels for stress recovery. Notably, there remains a research gap in China regarding assessment of plant species richness on stress recovery effect.

Some studies suggest that higher biodiversity enhances well-being and stress recovery by providing greater sensory stimulation and perceived naturalness [32,33]. Contradictorily, other past research has reported that excessive visual complexity or dense vegetation can reduce comfort and hinder cognitive restoration due to perceptual overload or feelings of enclosure [34,35]. This inconsistency highlights the need for more nuanced research into how people perceive and emotionally respond to varying degrees of plant diversity in small-scale urban parks.

The impact of landscape type on psychological restoration has drawn a lot of attention from researchers in recent years. while specific configurations, such as tree-grass combinations, may enhance perceived tranquility [36]. For landscape types, several studies on their restoration [37–39]. A past study demonstrated that tree–shrub–grass composite woodland has the best emotional recovery effect on students [33]. However, the findings only focus on students, lack more samples to analyze the recovery effect. In addition, studies of landscape type and restoration are most focus on large environments, such as forest [40,41], Few studies have investigated the relationship between landscape type and stress recovery in pocket parks. Hence, understanding landscape types is essential for optimizing green spaces to support their stress recovery, particularly in urban environments where access to nature is often limited. Moreover, people interpret the environment through different sensory [42–44], but it is unclear how people’s sensory perceptions of different landscape types affect stress recovery through their aesthetic perceptions. In conclusion, in the field of pocket parks, the evidence of the effect on different landscape types on stress recovery is seldom discussed [45].

### 2.3 Aesthetics of landscapes in pocket park

Landscape aesthetics constitutes an important field of landscape design and environmental psychology, emphasizing the perceptual and psychological effects of nature’s interaction with humans. Through aesthetic perception, people can experience restorative effects that reduce stress and enhance mental health. The importance of nature aesthetics has also been acknowledged in recent research on the connection to nature [46,47]. Aesthetic quality—such as color, shape, texture, and the overall visual appeal of natural elements—have been shown to stimulate positive emotional responses and elevate mood, particularly in high-density urban environments where natural beauty is scarce [17,48]. Several previous studies have shown that the diversity of plant communities has a significant positive impact on people’s aesthetic preferences [48–50]. Findings from past studies suggest that people’s visual aesthetics may function as a mediating mechanism between plant landscapes and stress recovery. However, there is a lack of studies focused on plant species richness and aesthetics of plant landscapes in pocket parks.

## 3. Methods

### 3.1 Study site

**Fig 1:**
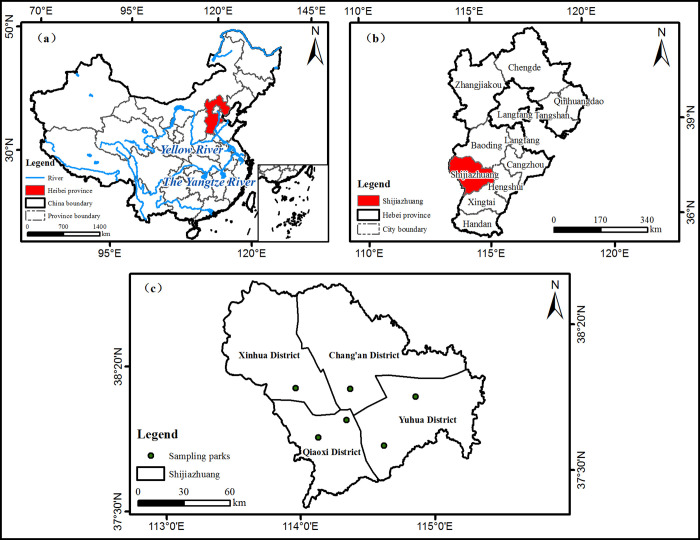
Study sites for this research. (a) Location of Hebei Province in China; (b) Location of Shijiazhuang in Hebei Province; (c) Location of the Six Parks in Shijiazhuang. Figure Source: generated by the first author using ArcGIS Pro software. The base map data were obtained from Natural Earth (https://www.naturalearthdata.com/).

This study was conducted in Shijiazhuang City, Hebei province of China (Fig 1). It’s a provincial city of Hebei province. Shijiazhuang has a total area of 14,530 square kilometers, a resident population of 11,233,500, and the urbanization rate of 72.28% [51]. As the capital city of Hebei Province, Shijiazhuang has experienced rapid urbanization in recent years. The increased population density and faster pace of life during urbanization have had significantly impacted the mental health of residents. Moreover, Shijiazhuang City has actively promoted the construction of urban green space in recent years. This is especially the planning and implementation of pocket parks. These pocket parks, because of their moderate scale and decentralized layout, have become an important place for urban residents to contact with nature on a daily basis. According to the Shijiazhuang Municipal Bureau of Parks and Gardens, a total of 665 pocket parks has been established across the city [52].

A purposive sampling strategy was adopted to select six pocket parks in Shijiazhuang, China. based on ‘Urban Green Space Classification Standard’ issued by the Ministry of Housing and Urban-Rural Development of China (2017) [53] and fulfill the purpose of this study, there are two criteria were used to select the pocket parks: (1) park type: according to the land use, pocket parks are classified into residential type, commercial type, and public serviceability type. In this study, to more accurately obtain the views and perceptions of urban residents, only residential type was selected for the research site. Residential pocket parks are typically situated within or near residential areas and cater to daily visitors from nearby communities, making them suitable for studying frequent user engagement and psychological recovery effects. In addition, the residential type has a relatively high visitation rate. Hence, the residential type of pocket parks should be chosen as the sample in order to more accurately capture the perceptions and experiences of urban residents; (2) park scale: On the basis of the Department of Housing and Urban-Rural Development of Hebei Province, pocket parks can be divided into three different scales by area. Small pocket parks are 400m^2^-2000m^2^ in size. Medium pocket parks are sized 2000 m^2^-5000 m^2^, large pocket parks are sized 5000 m^2^-10000 m^2^. Therefore, according to this criterion, pocket parks of three sizes, small, medium and large were included. This allows for more comprehensive information about participants in pocket parks of different scales. Through the above criteria, a total of six residential type pocket parks of Shijiazhuang city (Fig 1) were identified in this study, includes two small, two medium, and two large pocket parks, representing the diversity of park scales and allowing for a comparison of potential differences in stress recovery across sizes of pocket parks.

**Fig 2:**
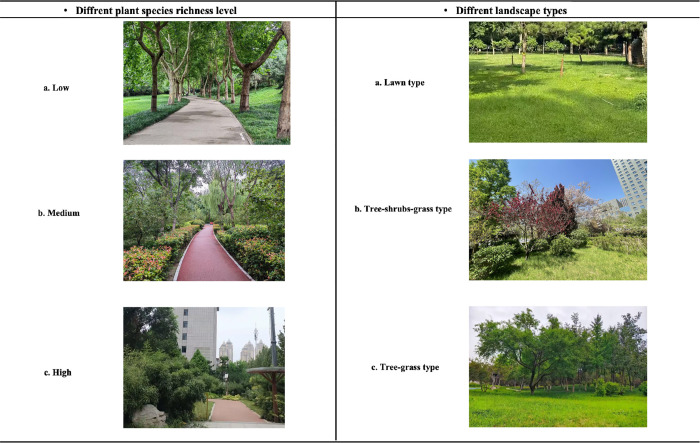
Images of plant species richness levels (low, medium, and high) and different plant landscape types (lawn, tree–shrub–grass, and tree–grass) in pocket parks. *Note*: Plant species richness was categorized three level, which includes low: < 5 species, medium: 5–10 species, high: > 10 species. Plant landscape type was categorized three types, which includes lawn, tree–shrub–grass, and tree–grass. Image source: The images were photographed by the first author.

Furthermore, plant survey was carried out to determine six pocket parks by quadrat survey. We used a random sampling method and selected 10m*10m areas within the park to record the number of plant species richness, which includes herbaceous plants, shrub and tree species. All vascular plants including naturally occurring vegetation and plant species were included to account for the full range of species richness visible to park visitors. Surveys were completed by two researcher and took approximately two hour per unit area. Based on previous empirical data [28] and because of the limitation of pocket park’s size, lower thresholds suitable for this study. Therefore, we categorized plant species richness as low (< 5 species) to medium (5–10 species) to high (>10 species). In addition, different landscape types have been identified based on a combination of trees, shrubs and grasses. Table 1 provides information of six selected pocket parks. The images for six typologies are provided in Fig 2 (Includes low, medium, high plant species richness areas and site with tree-shrub types, tree-shrubs-grass types and tree-grass types).

**Table 1:** The characteristics of six pocket park.

Scale	No.	Area	Describe of quadrat survey	Naturally occurring	Planted species
Small	1	1604 m^2^	With a low level of plant species richness (5 species)	1	4
	2	1950 m^2^	With a medium level of plant species richness (9 species)	3	6
Medium	3	4800m^2^	With a high level of plant species richness (17 species)	5	12
	4	4700m^2^	With tree-grass type	--	--
Large	5	9930 m^2^	With tree-shrubs-grass type	--	--
	6	7678.5m^2^	With lawn type	--	--

### 3.2 Participants recruitment

Participants are consisting of residents of Shijiazhuang. The participants were random sampling at the six selected pocket parks from January 10, 2025, to March 31, 2025. Through random sampling, every park visitor in the population has an equal probability of being selected as part of the sample [54]. Within each of the six selected pocket parks, participants were random approached in several typical activity zones—such as park entrances, seating areas, and walking trails—that represent common gathering and resting spots. This strategy aimed to capture diverse user experiences and perceptions across different spatial functions of the parks, rather than relying on a single random location. These zones ensured access to a diverse array of park visitors, increased the likelihood of capturing a representative sample across different demographics.

To ensure the validity of the study, participants were required to have no history of mental illness, not have consumed alcohol within twelve hours prior to the visitation, and not have cardiovascular disease or other factors that could interfere with the experiment [55]. Then, potential participants were asked if they were willing to complete a survey in this research.

This study was conducted in accordance with the Declaration of Helsinki, and the protocol was approved by the Ethics Committee of Taylor’s University (Reference No.: HEC 2024/441) and Institutional Review Board (IRB) of Hebei Institute of Communications (approval number: HMC102024). Informed consent was obtained in written form from all participants prior to their participation in the study. The anonymity and confidentiality of the participants were guaranteed, and participation was completely voluntary. We confirmed that all methods were conducted in accordance with the relevant guidelines and regulations.

### 3.3 Measurements and questionnaire design

There are two independent variables in this study: (1) perceived species richness; (2) perceived value of plant landscape type. The items for perceived species richness were adapted from prior studies. Selected items were based on their comprehensive approach to measure the plant diversity perception, items have been established validity in similar research contexts [56]. Five items were employed to measure plant diversity perception, such as “I perceive the plants of this park is richness and abundance”. Perceived value of plant landscape type refers to an individual’s perceived value and experiential assessment of specific plant landscape environments [57]. This study employed the perceived sensory dimensions (PSDs) scale [42] developed to measure the plant landscape type value. This scale shows a high reliability and validity to measure people’s perceived value of environments. The items of perceived value of plant landscape type are consisting of 8 items that aim to capture individuals’ experiences and perceived characteristic in plant landscapes, focusing on eight dimensions include “Natural”, “Cultural”, “Social”, “Serene”, “Diverse”, “Cohesive”, “Open”, and “Sheltered”.

The mediating variable in this study is perceived aesthetic quality. Perceived aesthetic quality is individuals’ subjective experience of the aesthetic properties of the environment through visual and emotional [17,18,58]. The perceived environmental aesthetic quality scale (PEAQS) [58] measures people’s perception of the aesthetic quality by six items. The PEAQS is a validated self-report tool that specifically measures key aesthetic dimensions within urban green spaces, making it particularly suitable for this research.

The stress recovery is the dependent variable of this study. The Restoration Outcome Scale (ROS) [59,60] was used to measure people’s stress recovery effect at different plant landscape sites. The scale is a self-evaluation of attention restoration for stress recovery. The ROS is a reliable and validated instrument for assessing the psychological benefits of restorative environments, which has been widely used to measure subjective restoration outcomes in environmental psychology studies [61,62]. The ROS scale has six items: three reflect relaxation and calmness, one reflects attention restoration, and two reflect clearing one’s thoughts.

Overall, the questionnaire for this study was divided into a pre-test and a post-test. The pre-test questionnaire consists of demographic information and construct of stress recovery. The post-test questionnaire consists of four potential constructs, include perceived species diversity, perceived value of landscape, perceived aesthetic quality and stress recovery. All scale items were used 5-point Likert scale (from ‘1 = strongly disagree’ to ‘5 = strongly agree’). The questionnaire items of each construct are shown in Tables 1 and 2 in S1 File.

**Table 2:** Respondents’ profile.

Demographic	Item	Frequency	Percentage (%)
Gender	Male	304	50.25
	Female	301	49.75
Age	18-30	187	30.91
	30-55	243	40.17
	56-69	146	24.13
	70 and above	29	4.79
Education level	High School	231	38.18
	Bachelor	272	44.96
	Master and above	102	16.86
Districts	Qiaoxi District	130	21.49
	Yuhua District	159	26.28
	Xinhua District	171	28.26
	Changan District	145	23.97
Monthly income	<3000R MB	170	28.10
	3000-5000 RMB	163	26.94
	5000-10000 RMB	202	33.39
	Above 10000 RMB	70	11.57
Visit frequency	1-3 time	89	14.71
	4-5 times	286	47.27
	6-10 times	197	28.22
	Over 10 times	59	9.74

### 3.4 Procedure

**Fig 3:**
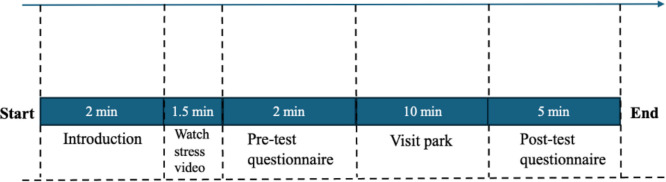
Data collection procedure.

The data collection process consisted of three phases: pre-test (baseline measurements), visit parks, and post-test (comprehensive assessment) (Fig 3), which took place in selected pocket parks in Shijiazhuang city.

Firstly, we briefly explained the survey purpose to participants. Next, we used videos to induce stress. Although video-based stress induction is a commonly used and ethically acceptable method in environmental psychology research, it has certain limitations that should be acknowledged. Such as the homogeneity of participants’ reactions to stress videos cannot be guaranteed. Nevertheless, videos showing accidents have been used as stressors with good results [63]. Specifically, we used a 1.5-minute compilation of urban traffic accident scenes [14]. Participants were initially exposed to a 1.5 min stress video to simulate pressure and mentally tense situations. Previous research has effectively used similar scenarios to induce mental stress [64,65]. To validate the effectiveness of video, a preliminary study involving 15 participants was conducted in which participants’ stress recovery were assessed using ROS before and after viewing the video [66]. The results showed a significant increase in stress, thus ensuring the validity of video-induced stress. After stress induced, participants completed a pretest questionnaire that included demographic information and ROS.

Then the participants were asked to visit the park in the study sites for around 10 minutes. A previous study noted that 10 minutes of exposure to a green environment significantly increased participants’ subjective well-being and task focus [66]. This study includes six sites with different landscape characteristics (low, medium, high plant species richness areas and tree-shrub types, tree-shrubs-grass types, and tree-grass types), every participant was visited only one sites. Each study site had a clear visual focus, which guided participants to distinguish between different landscape features.

In the third stage, the post-test aimed to collect comprehensive data regarding participants’ perceptions and experiences after exposure to the pocket park environment. To minimize other interferences, participants were invited to a quiet area within the park to complete the post-test survey. To reduce the impact of confounding variables, we have kept the surrounding environment was quiet and less noise. At the same time, the weather during data collection day was similar. The survey conducted on weekdays and weekends in the morning at 9:30–12:00 a.m., afternoon at 2:00–5:00 p.m., from January 10, 2025, to March 31, 2025. We distributed 678 questionnaires, 623 were returned, for a response rate of 90.1%. Out of the 623 questionnaires, 18 were excluded due to missing information. Thus, the valid questionnaires are a total of 605 (Male = 304, Female = 301). The respondents’ gender, age, educational attainment, districts, monthly income and visit frequency are shown in Table 2.

### 3.5 Data analysis

Questionnaire reliability was first tested by examining Cronbach’s alpha. The main data analyses included paired samples *t*-tests using SPSS (Version 27) and Structural Equation Modeling (SEM) using AMOS (Version 27) to assess relationships between variables. First, paired sample *t*-tests separately measured differences in stress recovery before and after in different landscape settings of pocket parks. Second, we developed Covariance-Based Structural Equation Modeling (CB-SEM), which emphasis on theory testing and its ability to assess the relationships between latent variables and observed indicators. The SEM was designed to evaluate relationships between the four latent variables: perceived species richness, perceived landscape value, perceived aesthetic quality and stress recovery.

Confirmatory Factor Analysis (CFA) was conducted to confirm the validity of the model, which included tests of convergent validity and discriminant validity. Model fit was evaluated using several indices: χ²/df, CFI (Comparative Fit Index) and TLI (Tucker-Lewis Index), RMSEA (Root Mean Square Error of Approximation). After ensuring the reliability of the questionnaire and the validity of the model, the path coefficients were analyzed to reveal the relationship between the four constructs. Finally, the bootstrap method with 5,000 resamples was used to analyze the mediating effects of perceived aesthetic quality.

## 4. Results

### 4.1 Reliability results

The reliability test results showed a high degree of questionnaire (Cronbach’s alpha > 0.7), ranged from 0.847 to 0.896 (Table 3 in S1 File). Therefore, the reliability of the questionnaire was ensured.

**Table 3:** Results of paired t-test analysis in stress recovery of pre-test and post-test with different plant species richness.

Pairing Group	Measure Item	*M*	*SD*	Mean difference	*t*	*p*	Cohen’s d
Low (n = 83)	Pre-test	2.15	0.56	−0.65	−7.098	0.000**	1.16
	Post-test	2.80	1.06				
Medium (n = 106)	Pre-test	3.82	0.51	−0.46	−10.502	0.000**	0.90
	Post-test	4.28	0.52				
High (n = 79)	Pre-test	3.39	0.84	−0.21	−2.375	0.019*	0.25
	Post- test	3.60	0.88				

*Note: p* = Significant level; * = *p < 0.05*, ** = *p* < 0.01; *M* = Mean; *SD* = Standard Deviation.

### 4.2 Effect of different plant species richness on stress recovery

We performed paired t-test on the variables of the pre-test and post-test on stress recovery. The results showed that there was a significant difference in low (t = −7.098, *p* < 0.01, Cohen’s d = 1.16), medium (t = −10.502, *p* < 0.01, Cohen’s d = 0.90) and high plant species richness level (t = −2.375, *p* < 0.05, Cohen’s d = 0.25) (Table 3).

### 4.3 Effects of different landscape types on stress recovery

As shown in Table 4, the paired sample t-test demonstrated that there were significant differences in stress recovery effect between three different plant landscape types. Specifically, the results showed that there was a significant difference in lawn landscape (t = −12.006, *p* < 0.01, Cohen’s d = 0.96), tree-shrubs-grass (t = −2.232, *p* < 0.05, Cohen’s d = 0.30) and high plant species richness level (t = −3.308, *p* < 0.0, Cohen’s d = 0.35).

**Table 4:** Results of paired t-test analysis in stress recovery of pre-test and post-test with different plant landscape types.

Pairing Group	Measure Item	*M*	*SD*	Mean difference	*t*	*p*	Cohen’s d
Lawn type (n = 122)	Pre-test	3.34	0.90	−0.76	−12.006	0.000**	0.96
	Post-test	4.09	0.67				
Tree-shrub- grass type (n = 116)	Pre-test	2.61	0.84	−0.28	−2.232	0.028*	0.30
	Post-test	2.89	1.03				
Tree-grass type (n = 99)	Pre-test	3.09	0.92	−0.36	−3.308	0.001**	0.35
	Post-test	3.45	1.11				

*Note: p =*Significant level; "* "= p < 0.05, "**" = p < 0.01; *M* = Mean; *SD* = Standard Deviation.

### 4.4 Structural equation modeling (SEM) analysis

#### 4.4.1 Validity of the model.

The model’s validity was first confirmed. We conducted confirmatory factor analysis (CFA) to test the proposed model’s validity, the analyzing includes: (1) Test internal consistency via Composite Reliability (CR); (2) Test convergent validity via Average Variance Extracted (AVE), and (3) The square root of the mean AVE value was calculated and compared with the correlation coefficients between variables to determine discriminant validity. As shown in Table 5, CR values ranged from 0.871 to 0.898, indicating good internal consistency, and the factor loadings were ranged from 0.673 to 0.798, which were all greater than recommended threshold of 0.5. The AVE value from 0.508 to 0.595, all AVE values are higher than 0.5. Overall, the convergent validity of the measurement model is adequate.

**Table 5:** Results of confirmatory factor analysis.

Constructs	Indicators	Items	Standard factor loadings	AVE	CR
Perceived Species Richness (PS)	Color diverse	PS 5	0.738	0.529	0.871
	Vegetation cover and density	PS 4	0.716		
	Native species richness	PS 3	0.758		
	Shrub richness	PS 2	0.712		
	Tree richness	PS 1	0.724		
Perceived Value of Plant Landscape Type (PV)	Sheltered	PV 8	0.71	0.508	0.892
	Open	PV 7	0.673		
	Cohesive	PV 6	0.674		
	Diverse	PV 5	0.712		
	Serene	PV 4	0.723		
	Social	PV 3	0.704		
	Cultural	PV 2	0.759		
	Natural	PV1	0.744		
Perceived Aesthetic Quality (PA)	Tidy	PA6	0.708	0.563	0.886
	Designed	PA5	0.748		
	Maintenance	PA4	0.745		
	Colorful	PA3	0.768		
	Mystery	PA2	0.756		
	Visual	PA1	0.776		
Stress Recovery (SR)	Clearing random thoughts	SR6	0.754	0.595	0.898
		SR5	0.759		
	Attention restoration	SR4	0.798		
	Relaxation and calmness	SR3	0.757		
		SR2	0.782		
		SR1	0.778		

In addition, the test of discriminant validity was good since the value on the diagonal (mean variance extracted AVE value) is higher than any of the correlation coefficients of the columns in which it is placed (Table 6). Furthermore, the fit indices indicated a robust fit to the model (χ² = 321.123, df = 293, χ²/df = 1.095; CFI = 0.998, TLI = 0.997, RMSEA = 0.010). The reference values and model fit results for the model fit indices are listed in Table 7.

**Table 6:** Discriminant validity test result.

Constructs	PS	PV	PA	SR
PS	**0.727**			
PV	0.383	**0.713**		
PA	0.519	0.355	**0.751**	
SR	0.489	0.387	0.627	**0.772**

*Note*: The bold numbers on the diagonal are AVE square root values.

**Table 7:** The reference values and model fit results for the model fit indices.

Fitting Index	Model Analysis Value	Critical Value	Interpretation
CMIN/DF	1.095	<3	Acceptable
NFI	0.961	>0.8	Acceptable
RFI	0.957	>0.8	Acceptable
IFI	0.998	>0.8	Acceptable
TLI	0.997	>0.8	Acceptable
CFI	0.998	>0.8	Acceptable
GFI	0.963	>0.8	Acceptable
RMSEA	0.010	<0.08	Acceptable

#### 4.4.2 Results of path coefficients.

Following the validation of the measurement model, the strength of the path coefficients was examined to ascertain that the loadings were significant in support of the theoretical assertions regarding what constituted each construct. Table 8 sheds light the interacted interplay between perceived species richness, perceived value of plant landscape type and perceived aesthetic quality and stress recovery. The results confirmed the significance of all hypothesized paths in the model at a significance *p* < 0.001.

**Table 8:** Model path coefficient estimation result.

Measurement paths	Estimate	S.E.	C.R.	*P*
SR < --- PS	0.185	0.055	3.385	0.000***
SR < --- PV	0.146	0.052	2.917	0.000***
PA < --- PS	0.448	0.056	8.681	0.000***
PA < ---PV	0.183	0.051	3.593	0.000***
SR < --- PA	0.479	0.056	8.766	0.000***

*Note: p =* Significant level; “***” = p < 0.001. PS: Perceived Species Richness; PV: Perceived Value of plant Landscape type; PA: Perceived Aesthetic Quality; SR: Stress Recovery.

**Fig 4:**
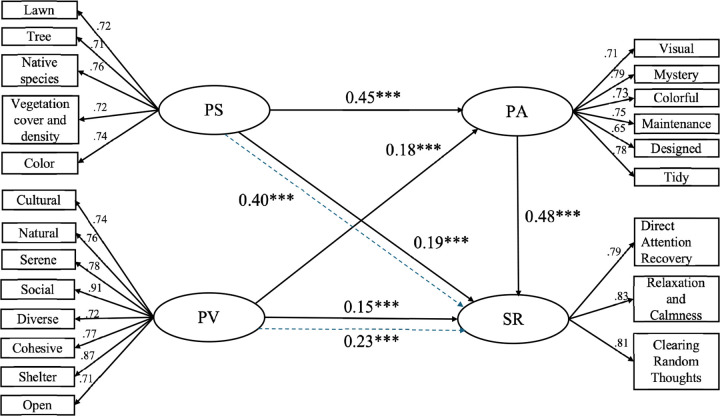
The structural model of the relationships among four variables and path coefficients results. Note: PS, PV, PA and SR, refer to “Perceived species richness”, “Perceived value of plant landscape type”, “Perceived aesthetic quality” and “Stress recovery”, respectively. The ellipse represents the latent variables, the square represents the observed variables. Two dashed lines with blue color indicate the indirect effect between the pairs of constructs via perceived aesthetic quality. *** = p < 0.001.

Specifically, a positive and significant directed effect was found between plant diversity perception and stress recovery (β = 0.185, *p* < 0.001), indicating that higher plant diversity perception directly contributes to stress recovery among residents. Likewise, a positive and significant directed effect was found between perceived value of plant landscape type and stress recovery (β = 0.146, *p* < 0.001), suggesting that residents with a stronger perceived value of plant landscape types experience better stress recovery. The strongest direct effect was observed between perceived aesthetic quality and stress recovery (β = 0.479, *p* < 0.001), this finding indicated the crucial role of aesthetics of plant landscapes in promoting people’s stress recovery. The structural model of the four constructs is shown in Fig 4.

### 4.5 Results of mediation analysis

In addition to direct effects, the mediating role of perceived aesthetic quality was analyzed using mediation analysis. We used Bootstrap method to test the mediation effect for PAQ by setting the sample to 5,000 times, and the confidence level of the interval was set to 95%, and biased-corrected confidence intervals were used as a basis for observing the upper and lower bounds. When the bias-corrected confidence interval for the indirect effect does not include 0, it means that there is a mediating effect. Perceived species richness exerted a significant indirect effect on stress recovery through its influence on aesthetic quality (β = 0.215, *p* < 0.001). Similarly, perceived value of plant landscape demonstrated a significant indirect effect on stress recovery mediated by perceived aesthetic quality (β = 0.088, *p* < 0.001). The total effect of plant diversity perception on stress recovery, accounting for both direct and indirect effects, was significant and positive (β = 0.400, *p* < 0.001). Likewise, the total effect of perceived value of plant landscape type on stress recovery, accounting for both direct and indirect effects, was significant and positive (β = 0.234, *p* < 0.001). The mediation analysis results are presents in Table 9.

**Table 9:** Mediation effect test results of the model.

Mediation paths	Type of effect	Efficiency value	Bootstrap 95% CI (n = 5000)		*p*
			LL	UP	
PS-- > PA-- > SR	Direct effect	0.215	0.155	0.292	0.000***
	Indirect effect	0.185	0.072	0.301	0.003*
	Total effect	0.400	0.310	0.498	0.000***
	Direct effect	0.088	0.042	0.144	0.000***
PV --- > PA --- > SR	Indirect effect	0.146	0.050	0.244	0.002*
	Total effect	0.234	0.141	0.327	0.000***

*Note:* *p* = Significant level; “*” = p < 0.05; “***” = p < 0.001. LL: Lower Limited; UP: Upper Limited; PS: Perceived Species Richness; PV: Perceived Value of plant Landscape type; PA: Perceived Aesthetic Quality; SR: Stress Recovery.

## 5. Discussion

### 5.1 Effect of plant species richness and landscape types on stress recovery

Plant species richness and landscape type in pocket parks were found to significantly influence psychological recovery. Specifically, medium level of plant diversity and lawn landscape type exhibited the strongest restorative effects. The result is consistent with Attention Restoration Theory [13], which suggests that the “attractiveness” and “compatibility” of natural environments can help reduce mental fatigue. For the influence of plant species richness on stress recovery, the findings of this study are similar to past research in other countries, such as the Nordic region, where plant species richness is considered a central factor in psychological recovery, while landscape design is more focused on integration with the natural landscape [36,42]. However, unlike a past finding advocated that a “higher diversity, higher recovery” perspective [31], the present study emphasized the importance of moderate diversity. A possible explanation for this is that moderate levels of plant species richness may provide sufficient visual appeal without reducing recovery due to information overload. Highly complex natural environments may reduce perceptual comfort due to increased cognitive load [67].

In addition, our results demonstrated that perceived species richness positively influence stress recovery, supporting findings from previous studies [33,34,68,69] showing that perceived plant species diversity contribute to restorative benefits. For instance, a recent study revealed that perceived biodiversity was positively associated with perceived restorativeness [33]. Meanwhile, perceived biodiversity often differs from actual biodiversity and tends to show a stronger association with human well-being, underscoring the importance of enhancing public awareness and recognition of plant species richness [34]. However, the findings of this study are contrary to the finding that higher perceived species richness is more likely to have higher negative mood states [70]. This discrepancy may be partly explained by methodological differences, particularly the use of virtual environments in prior research. In contrast, this study utilized pocket parks in real environments, where participants experienced the actual spatial of different plant species richness landscapes, potentially enhancing the restorative impact of perceived species richness.

Additionally, the medium level of plant species richness at the sites in this study exhibited richer plant colors within the area, which may have then led to participants perceiving higher plant diversity [71]. Also, due to the small differences in plant morphology across plant species richness levels in this study site, participants over- or under-estimated species richness [24,72]. In pocket park design, a rational layout of plant colors richness, and differentiate plants morphology can enhance visual appeal [17,48,73].

As for the effect of different plant landscape types on stress recovery, the results of the comparison of the stress restoration effects of the three landscape types were: lawn landscape > tree-grass landscape > tree-shrubs-grass landscape. Previous studies have suggested that viewing single-layer grassland has the best restoration effect, which is consistent with the results of this study. For example, several studies found that the environments with grass more strongly recovery people’s stress [62,63]. Likewise, another study pointed that lawn is a key factor increase he health and well-being [45]. A possible explanation is that lawn landscapes are often associated with openness and accessibility, which has been shown to reduce stress and promote relaxation [37]. This suggest that simpler and more open landscapes are more effective in facilitating stress recovery compared to more complex, multi-layered vegetation structures in pocket parks.

However, other studies demonstrated that viewing landscapes with lawn had a lower restorative effect [74]. This difference may be due to differences in objective factors such as plant species, plant growth across the study sites, resulting in different outcomes. Furthermore, in the unique culture background of China, lawns are associated with order, cleanliness, and open space—qualities that align with local aesthetic norms emphasizing harmony and balance [16]. Landscape aesthetics have historically emphasized balance, harmony, and order in Chinese cultural context [75]. Consequently, plant environments characterized by moderate plant species richness and open lawn landscapes may better align with these aesthetic values, enhancing emotional comfort and perceived restoration. Moreover, well-maintained lawns may evoke a stronger sense of safety and controllability, both of which are positively linked to psychological restoration [40,76].

Likewise, tree-grass landscapes also demonstrated a relatively strong stress recovery effect. This landscape may provide a sense of distance, satisfy the need for a lookout [37]. However, the lack of a shelter to provide protection does not fulfill the psychological need for shelter, thereby limiting the overall experience [77]. Notably, the tree-shrub-grass landscape proved to have the lowest stress recovery effect in this study, which contrasts with past research. While structural diversity can enhance engagement, it may also become overwhelming if perceived as chaotic or unorganized [78]. This may explain the lower restorative effect associated with tree–shrub–grass landscapes observed in this study. Overall, the findings demonstrates that different plant landscape types exert varying effects on stress recovery, although all landscape types examined positive stress recovery effects.

### 5.2 Impacts of plant species richness and vegetation types on aesthetics

Regarding the effect of plant species richness on aesthetics, the relationship is multifaceted. Most studies show that higher species richness is associated with greater visual appeal and aesthetic value of green space [71,79–81], supporting this study’s findings. For example, a study suggested that plant diversity interacts with structural arrangement to maximize aesthetic benefits, particularly in compact urban green spaces [81]. Simultaneously, another study argued that flower color diversity, combined with species richness, optimizes aesthetic preferences by adding vibrancy and seasonal variation to urban landscapes [17]. Therefore, incorporating flowering plants with distinct seasonal cycles can enhance aesthetic dynamics and user engagement.

Conversely, a previous study in Sweden [82], has found that high plant diversity in dense urban settings may lead to cognitive overload, thereby reducing aesthetic appreciation. This contrasts with the current study, which identified plant species richness was positively related to perceived aesthetic quality. The divergence may be explained by differences in spatial context, as pocket parks’ landscapes require simpler designs to avoid overwhelming users.

Furthermore, the results of this study emphasized a positive relationship between the perceived value for landscape types and aesthetic quality. Different landscapes play an important role in shaping aesthetic perceptions. Landscapes with clear and organized layouts are more likely to evoke positive aesthetic perceptions. For example, an empirical study noted that scattered trees combined with open grassy areas can create a pleasing and functional environment [83]. In addition, lawns are highly valued for their simplicity and coherence, aligning with the prior research [84], which highlighted that the neatness of lawn landscapes fosters relaxation and visual appeal. Perceived sensory dimensions offer a nuanced way to measure the aesthetic value of landscape types, This study reinforces the importance of integrating perceived value of plant landscape types in pocket park design to create aesthetically pleasing and restorative environments.

This study suggests a balance between the plan species richness and landscape types, which influence visitors’ aesthetic preferences. While people support biodiversity, neat and tidy spaces also shape aesthetic preference [85]. However, lawns are often associated with reduced herbaceous plant diversity due to intensive management practices. Moreover, a study from Japan suggested that public conservation preferences can vary by green space types and spatial scales [83]. Despite the limited species diversity of lawns, their restorative potential can be significantly enhanced through appropriate design and management. In addition, a previous study proposed the concept of ‘alternative lawn landscapes’, in which the biodiversity of lawns is enriched by incorporating native flowers, shrubs, and other herbaceous plants while maintaining their clean appearance [86]. Collectively, this study underscores the importance of balancing biodiversity and tidiness in pocket parks to optimize psychological restoration. Urban planners should adopt flexible planting layouts that combine open, tidy areas with visually rich and ecologically diverse zones.

### 5.3 Impact of aesthetic quality on public health and well-being

This study identified perceived aesthetic quality as a partial mediator in the relationship between plant landscape components and stress recovery. The outcome is similar with previous findings, which found the plant landscapes aesthetically pleasing were more likely to experience reduced stress, highlighting the critical role of subjective aesthetic perception in translating vegetation features into psychological benefits [87,88]. The results suggest that designers should pay attention to improve the perception of plant species richness through the vegetation layout, color contrast, and morphological combinations when planning green spaces. Aesthetic perceptions of green spaces significantly influence mental health and well-being by enhancing emotional states, reducing stress, and fostering cognitive restoration [45].

By fostering stress reduction, cognitive restoration, aesthetically pleasing landscapes contribute to people’s well-being. Therefore, vegetation landscape aesthetic quality in pocket parks should be prioritized in planning to promote public health. Thus, this study may contribute to plant selection and spatial arrangement, which prioritize structured yet naturalistic compositions, combining lawns, shrubs, and seasonal flowering plants to ensure visual order and year-round appeal [24]. Pocket parks that incorporate native or low-maintenance plant species [15] can reduce management costs and enhance ecological resilience.

## 6. Limitation of the study

Several limitations in this study must be taken into consideration. Firstly, the respondents’ stress recovery relied on self-reported measurements and short-term exposure for long-term wellbeing, which may be influenced by individual variability and recall bias. Future studies should incorporate objective indicators, such as heart rate, blood pressure, or skin conductance, and longitudinal research designs to enhance the reliability and robustness of the findings [89,90]. Secondly, although random sampling was applied among park visitors, the findings primarily reflect individuals who actively use pocket parks and may not fully represent city residents who seldom visit such spaces. Another limitation lies in seasonality. Data collection was conducted during a single season (spring–early summer), when vegetation was in full growth and environmental conditions were generally favorable. Seasonal variations in plant phenology, color, and user behavior may influence both aesthetic perception and restorative outcomes. Therefore, future studies should consider longitudinal or multi-seasonal measurements to capture temporal dynamics in restoration experiences. In addition, while the stress-induction method was carefully designed and preliminarily validated through a pilot study(n = 15), future research could strengthen this aspect by using larger samples and objective physiological measures. Moreover, the park sample may not fully represent other types of urban pocket parks (e.g., commercial, cultural, or ecological parks) or cities with different climatic, cultural, or spatial characteristics. Therefore, the conclusions primarily apply to similar residential urban environments rather than all pocket parks or urban green spaces.

Additionally, cultural differences may significantly influence individuals’ perceptions of plant landscapes and their stress restorative effects. For example, preferences for certain plant types or aesthetic features might vary across cultural [80] or geographical contexts, potentially limiting the generalizability of the findings to other regions or countries. Future studies could adopt cross-cultural approaches to explore whether similar patterns hold in diverse cultural settings. Finally, since our research comes from an on-site field in the real environments, it is inevitable that we will receive interference from uncontrollable factors, such as weather and noise. Including these factors as covariates in future studies or conducting controlled experiments can help minimize their impact. Overall, these limitations do not diminish the overall validity of the findings, but they highlight areas for future research.

## 7. Conclusion

As an important green spaces in high-density urban environments, pocket parks should be carefully planned. The focus should be on plant species richness and an optimal combination of lawn landscapes. These landscape features not only significantly reduce residents’ stress but also enhance the overall aesthetic quality and provide urban residents with an efficient place for stress recovery. These findings contribute to the theoretical understanding of restorative environments by extending Stress Recovery Theory (SRT) and Attention Restoration Theory (ART) to the context of pocket parks. Furthermore, this study has relevant implications for landscape specialists regarding the design and management of urban pocket parks in China and other parts of the world. The results clearly highlight the importance of balancing plant species richness with visual coherence, suggesting that moderate diversity enhances perceptual comfort and emotional restoration. Ultimately, improved management of plant landscape types has the potential to promote the psychological response for the city residents [33].

At the policy level, the findings emphasize the importance of recognizing pocket parks as critical components of urban green infrastructure that contribute to residents’ mental well-being and ecological health in China. Municipal authorities should promote the use of native species [91], incorporate aesthetic evaluation and user feedback mechanisms, to ensure that pocket parks are inclusive, culturally resonant, and environmentally resilient.

Based on the results of this study, specific design recommendations are proposed: (1) Medium plant species richness should be prioritized in the plan to provide a moderate level of visual complexity and cognitive comfort. By introducing native plant species, both ecological adaptability and residents’ aesthetic needs will be enhanced. In addition, enhance public awareness of plant diversity through public education and other methods to increase the perception. (2) Optimize lawn landscape design: In pocket park landscape planning, there is a need to focus on some open landscapes such as lawn types. Planting low shrubs or flowers around the lawn to increase landscape hierarchy and visual appeal. Furthermore, arranging the structure of the plant landscape according to the plant species and growth state, avoiding overly complex landscape structures. (3) Enhance perceived aesthetic quality through plant combinations of color, morphology while avoiding over-complication. For example, select flowering plants that bloom in different seasons to provide year-round color variation and visual interest. In addition, incorporate plants with varying heights and forms to create depth and hierarchy. (4) Incorporate culturally significant plants and design elements to align with local traditions and aesthetics. (5) Planting should be organized into visually coherent patterns, such as grouping flowering plants by color or arranging shrubs and trees in structured layers. This approach may balance plant species richness with a tidy and organized appearance, thereby enhancing the aesthetic appeal of the pocket park.

In summary, these findings are particularly meaningful for landscape planners and pocket park managers in formulating effective plant landscape guidelines to promote people’s stress recovery in China. Our study provides valuable insights into urban planning and landscape design aimed at enhancing human well-being in urban environments.

## Supporting information

S1 File Survey questionnaire and reliability results. (PDF)
