# Supplementary material for: Assessing stress restorative potential of plant species richness and plant landscape types of pocket parks: The mediating role of aesthetic quality
Source: PLoS One. 2026 Feb 13;21(2):e0343001. doi: 10.1371/journal.pone.0343001 (PMC12904447; doi:10.1371/journal.pone.0343001)
Supplement: S1 File — (PDF) [file pone.0343001.s001.pdf]

S1 Appendix. Survey questions of questionnaire and reliability results

Questionnaire Basic information:

1. Gender

A: Male B: Female

2. Age

A:18-30 B: 30-55 C: 56-69 D: 70 and above

3. Education level

A: High school B: Bachelor’s degree C: Master’s degree and above

4. Disctrict

A: Qiaoxi district B Yuhua district C: Xinhua district D: Changan district

5. Monthly income

A: <3000 RMB B: 3000-5000RMB C: 5000-10000RMB D: Above 10000RMB

6. Visit frequency per month

A: 1-3 B 4-5 C: 6-10 D: >10

Table 1. The constructs and indicators of the questionnaire for pre-test

Basic information		
Stress Recovery (SR)	Directed attention recovery	SR1: I’m able to focus my attention and concentration.
	Relaxation and calmness	SR2: I feel calm.<br>SR3: I feel relaxed.<br>SR4: I feel anergy and enthusiasm.
	Clearing one's thoughts	SR5: I can structure my thoughts.<br>SR6: I forgot of the troubles in my life

Table 2. The constructs and indicators of the questionnaire for post-test

Latent Variables	Observed Variables	Items
Perceived species richness (PS)	Native species	PS1: I think this park has lots of native plant species.
	Trees diversity	PS2: I think this park have many trees.
	Shrubs diversity	PS3: I think this park have many shrubs.
	Vegetation cover and density	PS4: I think the vegetation cover and density is good in this park
	Color diversity	PS5: I think this park looks colorful
Perceived value of landscape type (PV)	Cultural	PV1: It is a place offering a sense of being in nature on its own conditions (opposite of artificial)
	Natural	PV2: It is a place with traces of human efforts
	Serene	PV3: It is a place with a sense of calm and tranquil
	Social	PV4: It is a place suitable for socializing
	Diverse	PV5: It is a place with diverse scenery
	Cohesive	PV6: It is a place with cohesion and unity sensory
	shelter	PV7: It is a place offering a sense of sheltered and protection
	Open	PV8: It is an open place with a great outlook
Perceived aesthetic quality (PA)	Visual	PA1: The plant landscape in this pocket park is attractive.
	Mystery	PA2: The plant landscape in this pocket park is interesting
	Colorful	PA3: The plant landscape in this pocket park looks colorful.
	Maintenance	PA4: The plant landscape in this pocket park looks cared for
	Design	PA5: The plant landscape in this pocket park looks designed.
	Tidy	PA6: The plant landscape in this pocket park looks tidy.
Stress Recovery (SR)	Directed attention recovery	SR1: After visited this place, my attention and concentration are increased
	Relaxation and calmness	SR2: After visited this place, I feel calmer
		SR3: After visited this place, I feel more relaxed
		SR4: After visited this place, I feel more anergy and more enthusiasm
	Clearing one's thoughts	SR5: After visited this place helps me structure my thoughts.
		SR6: After visited this place, I forgot of the troubles in my life

Table 3. Reliability Results of the Questionnaire

Variables	Items	Corrected item-total correlation	Cronbach's alpha if item deleted	Cronbach's alpha
Perceived Species Diversity	PS1	0.655	0.86	0.847
	PS 2	0.681	0.856	
	PS 3	0.693	0.854	
	PS 4	0.693	0.810	
	PS 5	0.711	0.851	
Perceived Landscape Value	PL1	0.645	0.886	0.896
	PL2	0.673	0.883	
	PL3	0.675	0.883	
	PL4	0.67	0.883	
	PL5	0.694	0.881	
	PL6	0.673	0.883	
	PL7	0.695	0.881	
	PL8	0.683	0.882	
Perceived Aesthetic Quality	PA1	0.701	0.869	0.890
	PA2	0.688	0.871	
	PA3	0.696	0.87	
	PA4	0.711	0.868	
	PA5	0.718	0.867	
	PA6	0.71	0.868	
Stress Recovery	SR1	0.732	0.867	0.888
	SR2	0.698	0.873	
	SR3	0.714	0.87	
	SR4	0.727	0.868	
	SR5	0.686	0.874	
	SR6	0.688	0.874	
